# Immunogenicity of NSDV GP38 and the role of furin in GP38 proteolytic processing

**DOI:** 10.1128/jvi.00537-25

**Published:** 2025-06-10

**Authors:** Caroline Bost, Filipe Tomaz, Luna C. Schmacke, Sven Reiche, Nabil G. Seidah, Torsten Steinmetzer, Martin H. Groschup, Axel Karger, Sandra Diederich, Kerstin Fischer

**Affiliations:** 1 Institute of Novel and Emerging Infectious Diseases, Friedrich-Loeffler-Institut, Federal Research Institute for Animal Health 39023 , Greifswald-Insel Riems, Germany; 2 Institute of Pharmaceutical Chemistry, Philipps University Marburg, Marburg, Germany; 3 Department of Experimental Animal Facilities and Biorisk Management, Friedrich-Loeffler-Institut, Federal Research Institute for Animal Health 39023 , Greifswald-Insel Riems, Germany; 4 Laboratory of Biochemical Neuroendocrinology, Montreal Clinical Research Institute (IRCM), affiliated to the University of Montreal 5598 , Montreal, Québec, Canada; 5 Institute of Molecular Virology and Cell Biology, Friedrich-Loeffler-Institut, Federal Research Institute for Animal Health 39023 , Greifswald-Insel Riems, Germany; University of Kentucky College of Medicine, Lexington, Kentucky, USA

**Keywords:** orthonairovirus, NSDV, CCHFV, GP38, furin, serology

## Abstract

Nairobi sheep disease virus (NSDV) is a tick-borne orthonairovirus, which is genetically related to Crimean-Congo hemorrhagic fever virus (CCHFV), and causes severe hemorrhagic gastroenteritis in infected sheep. CCHFV GP38, a cleavage product of the CCHFV glycoprotein precursor (GPC), has recently attracted attention: not only has GP38 been reported to elicit detectable anti-GP38 antibodies in CCHFV-infected patients, but anti-GP38 antibodies have also been shown to protect mice from lethal CCHFV challenge. While proteolytic cleavage of CCHFV GP38 has been described to involve the proprotein convertases furin and subtilisin/kexin-isozyme-1 (SKI-1), little is known about the processing of NSDV GPC, or the occurrence and immunogenicity of NSDV GP38 in infected sheep. Here, we provide the first evidence for the presence and immunogenicity of NSDV GP38 in infected sheep demonstrating seroconversion by the detection of anti-GP38 antibodies over the course of infection. To further characterize GPC processing *in vitro*, we investigated the impact of furin overexpression and the effect of a furin inhibitor on NSDV glycoprotein expression, cleavage, and viral infectivity. While virus infectivity remained unaffected, our results suggest that other proteases besides furin may play a role in the proteolytic processing of NSDV GPC at a cleavage site that remains to be explored. Taken together, our findings highlight the immunogenicity of NSDV GP38 in sheep and warrant further research into the similarities and differences in proteolytic cleavage between the glycoproteins of NSDV and other orthonairoviruses, such as CCHFV.

**IMPORTANCE:**

Nairobi sheep disease virus (NSDV) is a zoonotic orthonairovirus causing severe and often fatal hemorrhagic gastroenteritis in small ruminants. Its genetic relationship to human-pathogenic Crimean-Congo hemorrhagic fever virus (CCHFV) and striking similarities in the clinical picture between CCHFV-infected patients and NSDV-infected ruminants have led to the idea that NSDV could serve as a model organism to study CCHFV pathogenesis. However, knowledge on NSDV-host interactions has been limited. While CCHFV GP38 has recently attracted attention as vaccine candidate and possible virulence factor, the occurrence and role of putative GP38 in other orthonairoviruses has been unclear. This study provides first evidence for the presence and immunogenicity of NSDV GP38 in infected sheep. Furthermore, our data indicate that other proteases besides furin may be involved in the proteolytic cleavage of NSDV GPC. Future studies are needed to determine the proteases involved and to investigate the possible functional role of GP38 in NSDV pathogenesis.

## INTRODUCTION

Nairobi sheep disease virus (NSDV) and Crimean-Congo hemorrhagic fever virus (CCHFV) belong to the genus *Orthonairovirus* (1). Both viruses are zoonotic and tick-borne so that virus circulation generally depends on vector presence and distribution (2). Moreover, both viruses circulate in enzootic tick-vertebrate-tick cycles involving a variety of domestic and wild animal species including ruminants. These serve as a blood source for the tick vector and, depending on the particular virus, may develop disease or only seroconversion (3–5).

For CCHFV, symptom severity and extent upon virus infection are reported to differ greatly between humans and ruminants. On the one hand, CCHFV is the etiologic agent of the most important tick-transmitted viral disease in humans called Crimean-Congo hemorrhagic fever (CCHF), a severe hemorrhagic fever disease with case fatality rates of up to 30% in infected patients (6, 7). On the other hand, ruminants are susceptible to CCHFV infection and may act as an intermediate host but do not show clinical symptoms (8–10). In contrast, an infection with NSDV is known to cause highly lethal hemorrhagic gastroenteritis in naïve small ruminants with mortality rates of up to 90%, making NSDV the most relevant orthonairovirus of veterinary importance (3, 11, 12), while only a few human cases with mild symptoms have been reported to date (13–15). However, given the similarities in the clinical pictures between NSDV-infected sheep and CCHFV-infected human patients, NSDV has been suggested as a model organism for studying CCHFV pathogenesis (16, 17).

The genomes of CCHFV and NSDV consist of three segments of negative-stranded RNA. The small (S) segment encodes for the viral nucleoprotein (N) and the small non-structural protein (NSs), the medium (M) segment encodes for the glycoprotein precursor (GPC), and the large (L) segment for the viral polymerase. Generally, proteolytic processing of the nairoviral GPC by host proteases appears to be very complex compared to other bunyaviruses (18–20). Proteolytic cleavage of the CCHFV GPC has been studied more intensively over the last years and shown to result in the production of structural glycoproteins (Gn and Gc) (21, 22) as well as several secreted non-structural glycoproteins (i.e., GP38, GP85, GP160), which seem to be unique to orthonairoviruses (18). In contrast, details about the proteolytic cleavage and maturation of GPCs of other orthonairoviruses are largely unknown.

The comparison of the amino acid sequences of different orthonairovirus GPCs suggests that some of the steps in GPC processing as well as some of the cleavage products may be common to several tick-borne members of the genus (19). Recently, we provided the first evidence that the subtilisin/kexin-isozyme-1, also known as site-1 protease (SKI-1/S1P), plays a similar role in NSDV infectivity as observed for CCHFV (23), for which SKI-1/S1P has been demonstrated to be involved in proteolytic processing of the structural glycoproteins of CCHFV, thereby strongly affecting CCHFV infectivity (22, 24). Besides SKI-1/S1P, another important host protease known to be involved in CCHFV GPC processing is furin, which cleaves the precursor at a furin cleavage site consensus motif (RSKR↓ [aa 244–247 of the CCHFV IbAr 10200 sequence]) that is highly conserved among different CCHFV strains (18, 25). Cleavage of CCHFV GPC by both furin and SKI-1/S1P generates GP38, a glycoprotein of 38 kDa, whose role and function during viral infection have only been recently studied in more detail. Due to the absence of a transmembrane domain, GP38 has been described to be secreted either alone or linked to the N-terminal mucin-like domain (MLD) as part of the GP160/85 proteins (18, 21, 25). Moreover, GP38 has been reported to play a role in the intracellular localization and maturation of the envelope glycoproteins Gn and Gc and to be essential for the production of infectious virions (26).

Noteworthy, CCHFV GP38 has been described to elicit specific antibodies in convalescent CCHF patients (27). In addition, it has recently gained attention due to its reported immunogenicity in mice, which developed non-neutralizing antibodies that were shown to protect mice from lethal CCHFV challenge (27, 28). As there are currently no licensed vaccines available for the prevention of CCHF, GP38 has become one of the target antigens for CCHFV vaccine research. However, its functions and the mechanisms of protection are still poorly understood. Regarding other relevant orthonairoviruses such as NSDV, even less is known about the proteolytic processing of the GPC and possible cleavage products including their immunogenicity in infected animals like sheep.

In this study, we addressed the question of whether a GP38-like protein is processed during NSDV infection and explored the potential contribution of furin to proteolytic cleavage of NSDV GPC. Moreover, we studied whether NSDV GP38 is immunogenic and elicits an antibody response in NSDV-infected sheep and investigated the possible use of GP38 as a serological target antigen for future serological studies.

## RESULTS

### Expression of a recombinant GP38-like cleavage product from NSDV GPC

**Fig 1:**
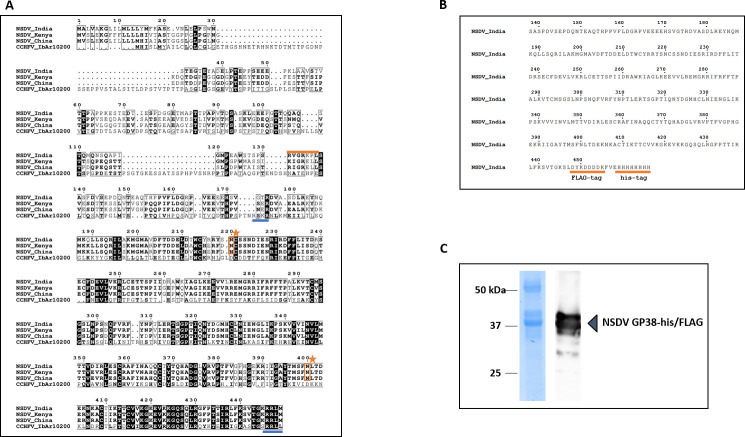
Production and characterization of recombinant NSDV GP38-his/FLAG. (**A**) Alignment of partial GPC sequences from different NSDV strains (Ganjam virus IG 619 = NSDV_India; NSDV 708 = NSDV_Kenya; NSDV H. longicornis China = NSDV_China) and CCHFV (IbAr10200). Numbering corresponds to NSDV_India GPC. Amino acid sequences are displayed starting from their respective N-terminus. For CCHFV, the furin protease cleavage site (RSKR) and SKI-1/S1P cleavage site (RRLL) are underlined in blue. For NSDV, arginine-containing motifs reported to be conserved across the GPCs of different orthonairoviruses are highlighted in orange. Two potential N-glycosylation sites (Asn_220_ and Asn_400_) are highlighted with an orange star. (**B**) For protein purification, the partial NSDV_India GPC sequence (aa 138–445; NSDV GP38-his/FLAG protein) was fused to a C-terminal 6×His- and FLAG-tag both highlighted with an orange underline. (**C**) SDS-PAGE of recombinant NSDV GP38-his/FLAG his-tag purified from Sf9 cells followed by Coomassie blue staining and immunoblot analysis using anti-FLAG primary and horseradish peroxidase-conjugated secondary antibodies.

To investigate the immunogenicity of a GP38-like protein during NSDV infection, we first aimed at expressing a recombinant NSDV GP38 for serological testing of sera from NSDV-infected sheep. In the absence of a classical consensus motif for recognition by furin and to identify the region within NSDV GPC that most likely resembled putative NSDV GP38, we first performed an amino acid (aa) sequence alignment of the N-terminal regions of CCHFV GPC (IbAr 10200) and the GPCs from Ganjam virus IG 619 (NSDV_India), NSDV 708 from Kenya (NSDV_Kenya), and an NSDV strain from China (NSDV_China). Based on this sequence alignment, a conserved recognition motif (RRLM) for potential proteolytic cleavage by SKI-1/S1P was identified at the C-terminal region of the alignment (Fig. 1A), which aligned with the C-terminal cleavage site of CCHFV GP38 (RRLL). Sequence design for the production of a recombinant NSDV GP38-like protein was based on different putative cleavage site recognition motifs present in the N-terminal region of NSDV_India GPC. Besides RVGR↓ (aa 131–134), the motif RKPL↓ (aa 134–137) had previously been described to be conserved across the GPCs of different orthonairoviruses including NSDV_India (19). Thus, we designed the resulting recombinant protein to cover the region of the NSDV_India GPC sequence between aa 138 (serine; S) and aa 445 (lysine, K). For efficient protein expression and purification from insect cells, the protein was fused to a C-terminal FLAG and 6×His--tag and is hereafter referred to as NSDV GP38-his/FLAG (Fig. 1B). The protein was successfully expressed and purified from insect cells (Fig. 1C). The removal of N-linked oligosaccharides by PNGase F treatment resulted in faster migration of the recombinant protein, suggesting the attachment of N-linked glycans to NSDV GP38-his-FLAG (Fig. S1).

### NSDV-infected sheep show seroconversion to recombinantly expressed NSDV GP38-his/FLAG

**Fig 2:**
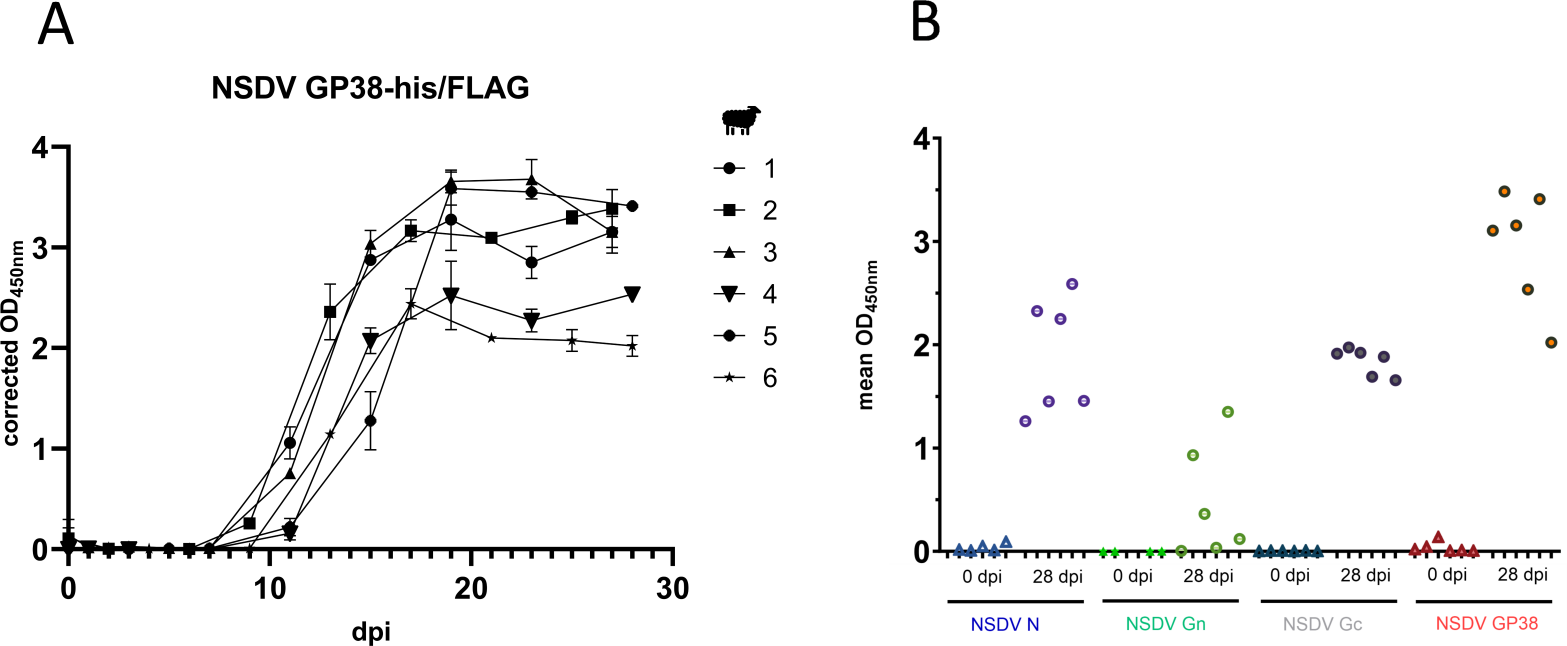
Seroconversion of NSDV- infected sheep against different NSDV antigens. (**A**) Sera from NSDV-infected sheep (*n* = 6) collected at different days post infection (dpi) were tested in duplicate in an indirect in-house ELISA based on recombinant NSDV GP38-his/FLAG. Mean of corrected OD values and standard deviations are displayed. (**B**) Serum samples collected from NSDV-infected sheep (*n* = 6) at 0 and 28 dpi were tested for reactivity in an indirect ELISA based on NSDV N-his/FLAG, commercially available recombinant Gn and Gc proteins, and GP38-his/FLAG.

The finding that convalescent CCHF patients developed anti-CCHFV GP38 antibodies during infection (27, 29) prompted us to investigate the potential humoral response of NSDV-infected sheep against putative NSDV GP38. Therefore, we first developed an indirect ELISA based on the recombinant NSDV GP38-his/FLAG and tested sera from sheep experimentally infected with NSDV (12). Seroconversion to recombinant NSDV GP38-his/FLAG was observed in all sheep starting around 8 dpi (Fig. 2A). We also examined the antibody responses of six sheep to the nucleoprotein (N; NSDV N-his/FLAG) and to two commercially available glycoproteins (NSDV Gn and Gc; Fig. 2B) using only serum samples collected at 0 dpi and 28 dpi. All sheep showed seroconversion to recombinant NSDV GP38, N, and Gc at 28 dpi (Fig. 2B). Interestingly, only three of six sera from NSDV-infected sheep exhibited a strong reaction to the Gn antigen. Overall, these findings suggest that GP38 is present and immunogenic in NSDV-infected sheep and, thus, may be suitable as a target antigen for future serological studies.

### *In vitro* characterization of NSDV GP38 during infection

**Fig 3:**
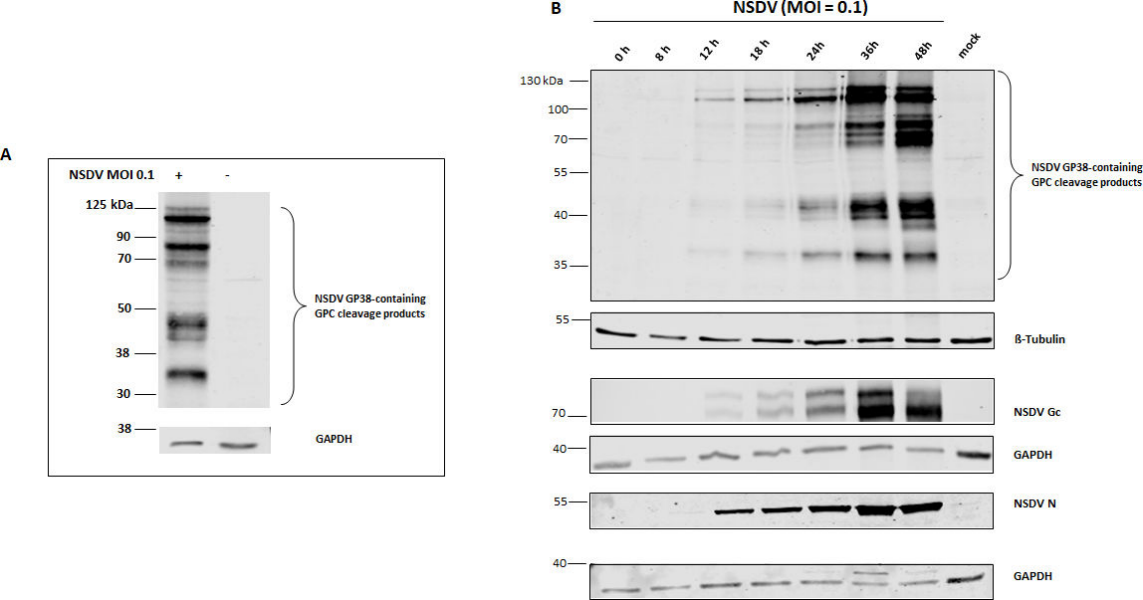
Immunoblot analysis of NSDV protein expression in SW13 cells. (A) Immunoblot analysis of NSDV-infected and mock-infected SW13 cells collected at 28 h p.i. For detection, newly generated monoclonal antibody (mAb) 6F6 A3B raised against NSDV GP38-his/FLAG and goat anti-mouse IRDye 800-conjugated antibodies were used. The detection of GAPDH served as a loading control. (B) Cell lysates from multi-cycle infection kinetics were collected at the indicated time post infection (p.i.). For detection, mAb 6F6 A3B and mAb 5H11 C1 (raised against NSDV Gc) and rabbit-derived polyclonal serum (R8253) for nucleoprotein (N) detection were used as primary antibodies followed by incubation with goat anti-mouse or anti-rabbit IRDye 680 or 800CW-conjugated secondary antibodies, respectively. β-Tubulin and GAPDH served as loading controls. Representative blots from three independent experiments performed in duplicates are shown.

To further investigate whether a GP38-like protein is processed during NSDV infection, we next generated specific monoclonal antibodies (mAb) for *in vitro* use. For immunization of mice, we used the recombinantly expressed NSDV GP38-his-FLAG and obtained a potent mAb (6F6 A3B) that was shown to specifically detect NSDV GPC in indirect immunofluorescence assay (iIFA; Fig. S2) as well as GPC cleavage products in immunoblot of NSDV-infected human adrenocortical carcinoma cells, also known as SW13 cells (Fig. 3A). Interestingly, 6F6 A3B detected at least four distinct bands whose molecular weight estimation by Empiria Studio 3.2 software revealed the approximate sizes of ~37 kDa, ~49 kDa, ~87 kDa, and ~113 kDa, of which the detected protein band with the lowest molecular weight, i.e., ~37 kDa, could represent the putative NSDV GP38. Linear and conformational epitope mapping of the mAb 6F6 A3B performed by Biosynth (Netherlands) as described in references 30, 31 revealed binding of the mAb 6F6 A3B to the epitope QLLSQRILAKMGMA (aa 189–202) of the NSDV GPC.

To study the expression and processing of NSDV GPC, lysates of SW13 cells infected with NSDV and collected at different time points (0, 8, 12, 18, 24, 36, and 48 h post infection; p.i.) were analyzed by immunoblot using mAb 6F6 A3B as well as an anti-NSDV Gc mAb (5H11 C1), and NSDV N-specific antibodies. NSDV N was readily detectable starting from 12 h p.i., while NSDV Gc, GP38, and other GPC cleavage products were visible but still of weak intensity at that time (Fig. 3B). A strong increase in band intensities was observed for N and GPC cleavage products with the strongest band intensities detected at 36 and 48 h p.i. (Fig. 3B).

### Furin overexpression increases NSDV GP38 cleavage *in vitro*

**Fig 4:**
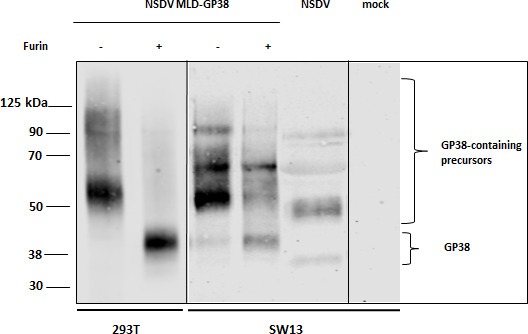
NSDV GP38 expression in the supernatant of transfected or NSDV-infected cells. HEK 293T and SW13 cells were transfected with pcDNA NSDV MLD-GP38-Strep (−) or co-transfected with pcDNA NSDV MLD-GP38-Strep and pIR-hfurin encoding for human furin protease (+). Supernatants were harvested at 72 h post-transfection and analyzed by immunoblot using mAb 6F6 A3B. For comparison of NSDV GPC cleavage products, supernatant from NSDV-infected SW13 cells (24 h p.i.) was also analyzed by immunoblot. A representative blot from at least three independent experiments is shown.

Given the similarities observed between CCHFV and NSDV in SKI-1/S1P protease usage (22, 23), we wondered whether NSDV GPC processing would equally depend on furin cleavage despite the lack of an optimal consensus motif for furin. We, thus, designed a plasmid encoding the first 445 amino acids of NSDV GPC fused to a Twin-Strep-tag at the C-terminus for purification (pcDNA3.1 NSDV MLD-GP38-Strep). This sequence has been designed in accordance with the approach described for the recombinant expression of CCHFV GP38, which, besides the actual GP38 sequence, also includes the N-terminal mucin-like domain (MLD) and the native furin cleavage site (RSKR; aa 244–247 in the GPC sequence of CCHFV Ibar 10200) for proteolytic processing of GP38 (32–34). Purification of Strep-tagged NSDV proteins from the supernatant of transfected HEK-293T cells demonstrated the presence of a protein band of ~51 kDa as detected in immunoblot analysis (Fig. 4), confirming the processing of the construct and secretion of the protein to the supernatant. To investigate the effect of increased furin expression on proteolytic cleavage, furin was transiently overexpressed by co-transfection. Remarkably, under these conditions, we observed a significant increase in detected intensities for a ~40 kDa protein, while the intensity of the ~51 kDa protein band was strongly reduced (Fig. 4), indicating an increased cleavage of the ~51 kDa precursor. The same experiment was repeated in SW13 cells, which serve as a cell line known to be highly susceptible to NSDV. Similarly, furin overexpression increased the amount of putative NSDV GP38, while the band intensity of the ~51 kDa precursor was strongly decreased (Fig. 4). Importantly, immunoblot analysis of the supernatant of NSDV-infected SW13 cells harvested at 24 h p.i. revealed the presence of multiple cleavage products with at least two similarly sized proteins (Fig. 4). The small increase in molecular weight of the transiently expressed recombinant proteins in contrast to the viral proteins detected in the supernatant of infected cells likely corresponds to the Twin-Strep-tag, which is reported to be ~2–3 kDa in size. Together, these data provide first evidence that NSDV GP38 and the respective precursor are, indeed, expressed and secreted into the supernatant of both transfected and infected cells. Furthermore, our findings indicate that the efficiency of NSDV GP38 cleavage can be increased by furin overexpression.

### NSDV GP38 is N-terminally cleaved at an uncharted cleavage site

To determine the N-terminus of NSDV GP38, samples of GP38 protein purified from HEK-293T cells transiently overexpressing furin were analyzed by SDS-PAGE (35), and bands corresponding to the processed GP38 protein at approximately 40 kDa were excised (Fig. S3A). After in-gel digest with Lys-C or trypsin, respectively, no peptides were identified mapping to the N-terminus of the GP38 precursor up to amino acid 175, suggesting the processing of the precursor protein at this position (Fig. S3B). As this site is also a tryptic cleavage site of the GP38 precursor protein, Lys-C generated and tryptic peptides were analyzed in detail. In contrast to trypsin, Lys-C does not cut the C-terminal of arginine residues (36) so that the Lys-C-generated peptide DVASDLREYNQMK is likely to represent the N-terminus of the processed GP38 precursor at position 176. The corresponding putative N-terminal tryptic peptide DVASDLR spanning from position 176 to 182 of the precursor was not detected after Lys-C digest, as expected, but was present in the tryptic digest. Also, no peptides corresponding to another potential cleavage site 134↓135 with two arginine residues further upstream (RVGR; aa 131–134) were detected neither using semi nor full enzyme specificities. Fragment spectra of the mentioned peptides are given in Fig. S3C.

**Fig 5:**
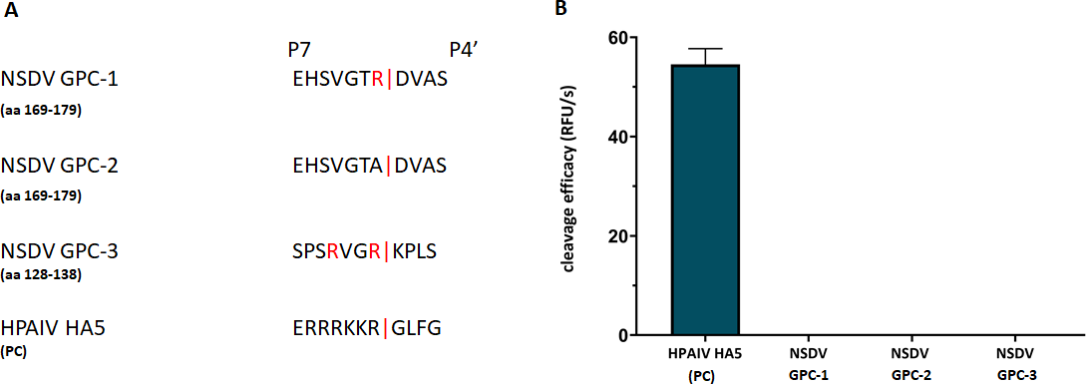
Efficiency of cleavage of FRET substrates by furin. (**A**) FRET substrates spanning the P7-P4′ amino acid sequences of the putative cleavage site motifs containing an N-terminal o-aminobenzoyl fluorophore and a C-terminal Tyr(3 NO_2_)-NH_2_ as a quenching residue were synthesized and tested in an enzyme assay with recombinant furin. Three NSDV GPC sequences were tested: GPC-1 covering the monobasic motif at position 175; GPC-2, a negative control for GPC-1 with the arginine of the motif replaced by alanine, and GPC-3 covering another putative dibasic furin motif for cleavage after residue 134. A FRET substrate with a multibasic furin cleavage site motif of hemagglutinin (HA5) of a highly pathogenic avian influenza virus (HPAIV) strain served as positive control (PC). (**B**) The cleavage of the FRET substrates by recombinant furin was measured in an enzyme kinetic assay. Mean values + SD based on three independent measurements with three independent weights of substrates are displayed.

Despite the absence of an optimal consensus motif for recognition by furin, we aimed to investigate the ability of furin to cleave the putative cleavage site as determined by mass spectrometry. Therefore, we designed and synthesized FRET substrates containing seven residues upstream (until position P7) and four residues downstream (until position P4′) of the putative monobasic cleavage site (aa 169–179; NSDV_India GPC; Fig. 5A, referred to as GPC-1; and GPC-2, with the arginine of the GPC-1 motif replaced by alanine as negative control). Another FRET substrate spanning aa residues 128–138 of the NSDV_India GPC sequence containing a potentially better suited dibasic segment with two arginine residues in positions P1 and P4 as a putative motif for recognition by furin (aa 131–134; motif RVGR, GPC-3) was also synthesized and included in the analyses (Fig. 5A). A FRET substrate with a classical multibasic furin cleavage site from an influenza H5 hemagglutinin (HA) served as positive control (Fig. 5A). While the positive control HA was efficiently cleaved by furin, no enzymatic activity was detected for any of the putative NSDV substrates (Fig. 5B).

### The furin inhibitor MI-1148 impacts the viral glycoprotein processing without affecting virus infectivity

**Fig 6:**
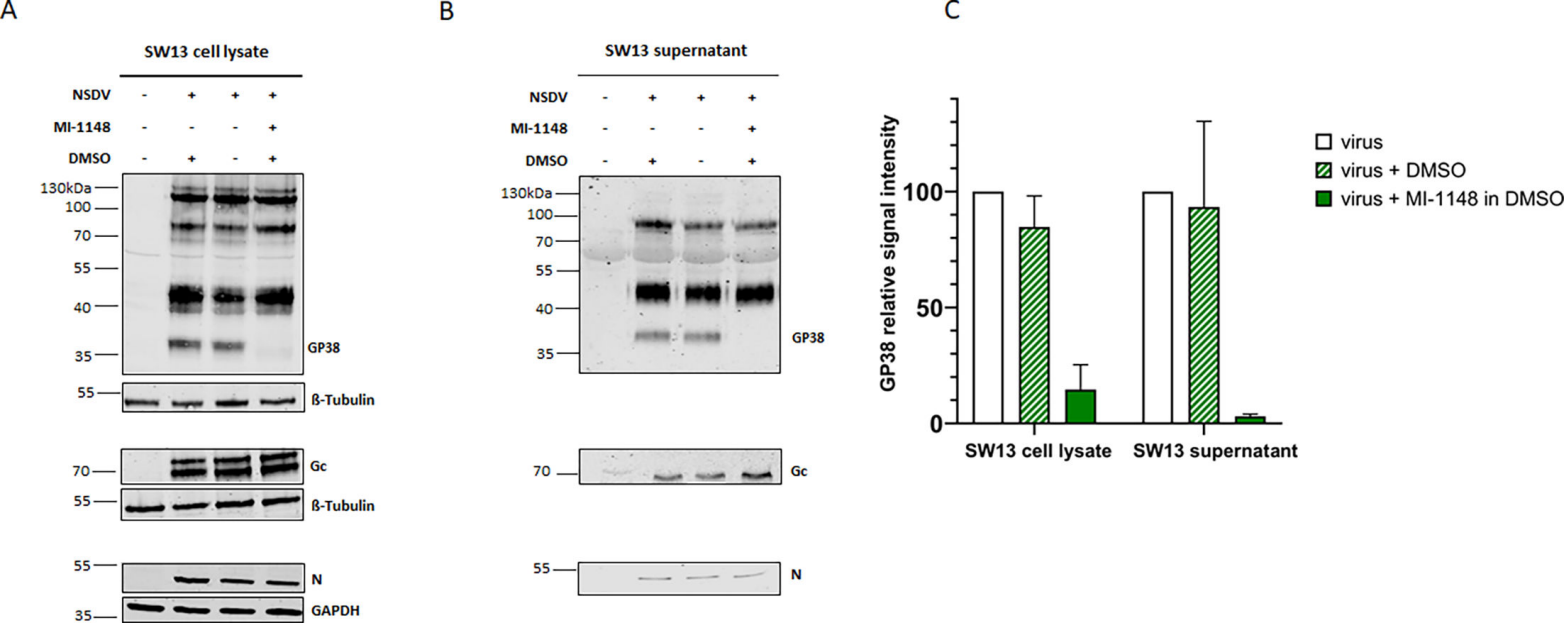
Impact of furin inhibition on NSDV glycoprotein processing. SW13 cells were infected with NSDV (MOI of 0.1). After removal of inoculum, fresh medium with (+) or without (−) furin inhibitor MI-1148 (30 µM in DMSO) or DMSO alone was added. At 24 h p.i., cell lysates (**A**) and supernatants (**B**) were collected and analyzed for N, Gc, and GP38 expression in immunoblot. β-Tubulin and GAPDH served as loading controls. Representative blots from three independent experiments each performed in duplicate are shown. (**C**) The signal intensities of the GP38 bands were quantified using Li-Cor software Image Studio Lite Ver 5.2. The GP38 signal intensities from inhibitor-treated samples were set in relation to the signal intensities of the untreated samples (=100%). Mean relative signal intensities and standard deviations from three independent experiments each performed in duplicate are shown.

Lastly, to study the effect of furin on NSDV GPC processing and infectivity, NSDV-infected SW13 cells were treated with the specific furin inhibitor MI-1148 (37). Prior to use, potential cytotoxic effects of inhibitor MI-1148 were evaluated. Cell viability of SW13 cells after 24 h or 48 h of inhibitor treatment with the indicated concentrations was not affected (Fig. S4). To investigate a possible effect of the furin inhibitor on proteolytic processing of NSDV GPC, we analyzed cell lysates and supernatants from NSDV-infected and inhibitor-treated SW13 cells by immunoblot. For NSDV Gc and N, no significant changes in signal intensities were observed between inhibitor-treated and untreated cell lysates or supernatants (Fig. 6A and B), indicating that their expression and processing were independent of furin activity. In contrast, the signal intensity of the GP38 cleavage product was strongly reduced in furin inhibitor-treated cells (Fig. 6A) as well as in the supernatant of MI-1148-treated cells (Fig. 6B). For quantification, GP38 signal intensities from infected cells in the absence of the furin inhibitor were set to 100%, and the signal intensities of GP38 from infected cells treated with MI-1148 were compared (Fig. 6C). The quantification showed a reduction of GP38 signal intensities of 85% and 97% in inhibitor-treated cells and supernatants, respectively.

**Fig 7:**
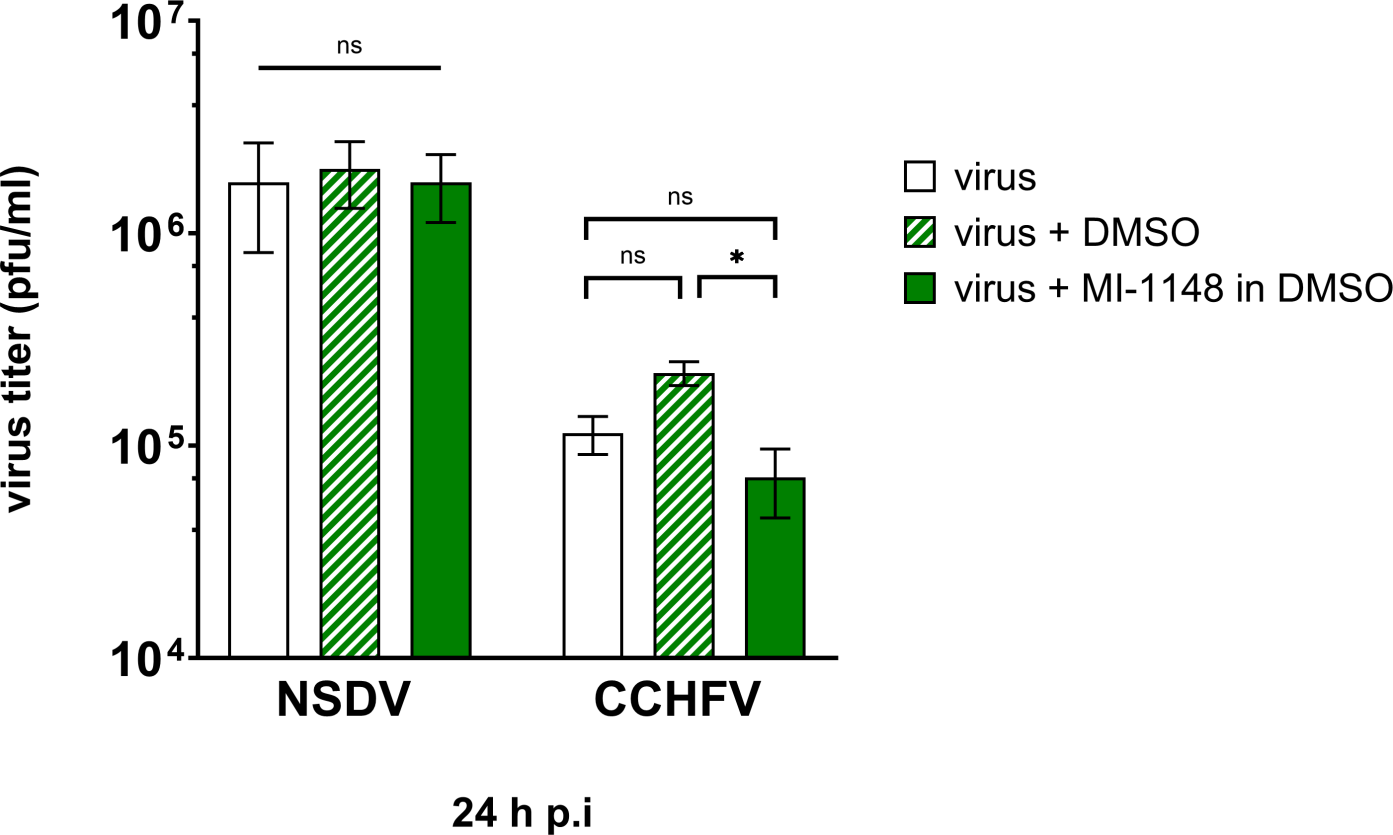
Impact of furin inhibition on NSDV and CCHFV infectivity. SW13 cells were infected with either NSDV or CCHFV at an MOI of 0.1. At 1 h p.i., inoculum was removed and fresh medium with (+) or without (−) MI-1148 was added in a concentration of 30 µM in DMSO for 24 h. DMSO-treated cells served as control. Virus titers were determined by plaque assay. Mean and standard deviation of virus titers are displayed from at least two independent experiments, performed in duplicate. Statistical analysis: unpaired *t*-test; (*) *P* ≤ 0.05; ns, not significant (*P* ≥ 0.05).

Therefore, inhibitor MI-1148 was added 1 h after NSDV inoculation, and cells were further incubated for 24 h. No significant differences in NSDV titers as determined by plaque assay were observed between the inhibitor-treated and -untreated cells (Fig. 7), indicating that the infectivity of NSDV is independent of furin activity. As a comparison, we performed the same experiment using CCHFV. Here, inhibitor treatment of CCHFV-infected SW13 cells resulted in a weak but significant change of viral titers of less than 1 log between inhibitor-treated and DMSO-treated cells (Fig. 7). As a control for the activity of the inhibitor, we tested the effect of MI-1148 on Measles virus (MeV), known to rely on furin cleavage for proteolytic activation of the fusion protein F_0_, which is a prerequisite of cell-cell fusion (38). As expected, no syncytia formation was observed in the inhibitor-treated cells (Fig. S5).

## DISCUSSION

Recently, a continuously growing number of studies has highlighted CCHFV GP38 as a promising vaccine candidate for protection from symptomatic CCHFV infection (27, 29, 32, 39) Klicken oder tippen Sie hier, um Text einzugeben. However, its role and functions as well as the mechanisms of action are still poorly understood. In this study, we aimed to shed light on the processing of NSDV GPC, as a closely related pathogen, and the presence and immunogenicity of GP38 or a GP38-like protein in NSDV-infected sheep.

To address this, we first expressed a recombinant NSDV GP38 using putative GPC cleavage sites as N- and C-terminal ends that had been previously predicted for NSDV (19). The recombinant NSDV GP38-his/FLAG antigen was used in ELISA to test serum samples from NSDV-infected sheep. In all samples analyzed, we observed a seroconversion of all the sheep to GP38, which provides the first evidence for the presence and immunogenicity of NSDV GP38 in sheep during NSDV infection. This is in line with the detection of anti-CCHFV GP38 antibodies found in convalescent CCHF patients (29) and demonstrates the importance of the so far understudied protein. For flaviviruses, it has also been described that a secreted non-structural protein, known as NS1, elicits specific antibodies in flavivirus-infected patients and animals (40, 41). Furthermore, NS1 has been widely demonstrated to contribute to flaviviral pathogenesis by disrupting endothelial barrier functions, influencing immune responses, and manipulating complement activation (42, 43). Interestingly, a very recent study has described a similar functional impact of CCHFV GP38 on endothelial cells contributing to vascular leakage and, thus, to viral pathogenesis (44). This is also consistent with the fact that non-neutralizing monoclonal antibodies raised against CCHFV GP38 were shown to protect mice against lethal CCHFV challenge (27, 28). While our findings confirm seroconversion of sheep and serum reactivity to recombinant NSDV GP38, future studies are needed to investigate its possible impact on NSDV pathogenicity in infected ruminants.

For CCHFV, the target of neutralizing antibodies is known to be Gc (29), which plays a key role in mediating CCHFV cell entry through its interaction with the recently identified low-density lipoprotein receptor (45). For NSDV, however, little is known about the glycoprotein-specific immune responses that contribute to virus neutralization (46). The serum samples from NSDV-infected sheep used in this study were previously reported to contain both anti-nucleoprotein (N) antibodies and NSDV-neutralizing antibodies (12). To further decipher antigen-specific reactivities, we compared antibody responses of these NSDV-infected sheep to recombinant GP38, Gn, and Gc as well as to N and found uniform seroconversion to all NSDV antigens except Gn. The observed partial lack in response to NSDV Gn could be due to the structural arrangement of Gn on the virus particle. A recent study on the glycoprotein complex of CCHFV has provided the first evidence that GP38 may be at least transiently associated with Gn and Gc on the viral surface and could thus mask Gn to a certain degree on the mature virion, which may result in lower levels of detectable anti-Gn antibodies (47). The lower abundance of anti-NSDV Gn antibodies observed in this study may indicate a similar structural arrangement of the NSDV glycoproteins. However, it remains to be investigated to what extent NSDV GP38 is associated with the virus particle and whether it circulates in the bloodstream of infected sheep as a secreted cleavage product.

While it is known that the GPCs of different CCHFV strains possess a highly conserved classical furin consensus motif (RSKR) at the N-terminal end of GP38 that is cleaved by furin (18), proteolytic processing by furin has not been described for any other orthonairovirus including NSDV. To date, only annotation and prediction tools have been applied (19). In this study, we used transient furin overexpression as well as furin inhibition by the potent substrate analogue furin inhibitor (MI-1148) (37) to gain first insights into the potential role of furin in the replication cycle of NSDV. Furin overexpression in human cells led to a strong increase in signal intensities for the GPC cleavage product GP38, while band intensities of the higher molecular weight precursors were clearly reduced. Moreover, inhibition of furin activity in NSDV-infected cells using the furin inhibitor MI-1148 led to a drastic decrease in the detection of NSDV GP38 in both cell lysates and supernatant, while higher molecular weight precursors were still detected. However, the potential N-terminus of NSDV GP38 identified by mass spectrometry in this study revealed an atypical site for direct furin involvement, which was supported by the inability of furin to cleave synthetic FRET substrates mimicking this sequence. Hence, the overexpression of furin as well as the inhibition of furin activity by MI-1148 may have a modulatory effect on other proteases possibly involved in GPC processing, and thus only have an indirect effect on proteolytic cleavage of NSDV GPC, which we did not further address in this study. Interestingly, experiments with a structurally very similar inhibitor containing Val as the P3 residue instead of tert. Leu (Tle) as the only difference suggests that MI-1148 may inhibit five of the seven basic proprotein convertases (i.e., furin, PC1/3, PC4, PACE4, and PC5/6) (48). In contrast, trypsin-like serine proteases as well as trypsin itself were significantly less inhibited (~10,000 times) by MI-1148 compared to furin (49).

However, regarding the release of infectious virus into the cell supernatant, the inhibitor treatment had no significant effect on viral titers of NSDV and only a weak but significant effect on CCHFV titers of less than 1 log. Even though the tools used cannot be compared completely, our findings are consistent with a previous study on CCHFV, in which only a transient early decrease in CCHFV viral titers was noted when grown on furin-deficient cells or when a cleavage-incompetent recombinant CCHFV was examined (25). Importantly, however, the biological activity of the inhibitor used in this study was confirmed in cells infected with MeV, a paramyxovirus highly dependent on proteolytic activation by furin (38, 50). Furthermore, MI-1148 has also been described to efficiently inhibit the furin-dependent proteolytic activation and thus the spread of different furin-dependent viruses including canine distemper and mumps virus, alphaviruses such as Chikungunya and Semliki Forest virus, flaviviruses like West Nile and Dengue virus, and highly pathogenic avian influenza viruses (37, 51–53).

Together, this is the first study to describe and characterize GP38 for the orthonairovirus NSDV. Our results suggest a similarity between the GP38 of CCHFV and NSDV in terms of immunogenicity in infected host species. The detection of anti-NSDV GP38 antibodies in serum from NSDV-infected sheep highlights the potential of this antigen for future serological studies where GP38 could complement existing assays. Regarding the role of furin in proteolytic cleavage, our findings indicate that NSDV GPC may differ from CCHFV, which warrants further studies to identify other proteases potentially involved in GPC processing.

## MATERIALS AND METHODS

### Cell lines and virus isolates

Human adrenocortical carcinoma (SW13) cells (kindly provided by Ali Mirazimi, National Veterinary Institute, Sweden) were maintained in Leibovitz-15 (L-15) medium supplemented with 5% fetal calf serum (FCS; L-15-5) and incubated at 37°C without CO_2_. Vero E6 cells (Collection of Cell Lines in Veterinary Medicine, FLI, CCLV-RIE 0929) and human embryonic kidney 293T cells (HEK-293T, FLI, CCLV-RIE 0197) were cultivated in Minimal Essential Medium (Earl’s and Hank’s salts 1:1) supplemented with 1% non-essential amino acids, 0.125% sodium hydrogen carbonate, 0.012% sodium pyruvate, and 10% FCS and incubated at 37°C with 5% CO_2_. *Spodoptera frugiperda* ovarian cells (Sf9, FLI, CCLV 203) were cultivated in Grace’s insect basal medium with 10% FCS and incubated at 27.5°C with 2.5% CO_2_.

The NSDV isolate (Ganjam virus IG619; hereafter referred to as NSDV_India; GenBank accession number: KU925466, KU925465, KU925464) and CCHFV isolate Kosova Hoti (GenBank accession number DQ133507, EU037902, EU044832) used in this study were propagated on SW13 cells. Measles virus (MeV, strain Edmonston, NR-3847) was propagated on Vero E6 cells. All work with live CCHFV was performed under BSL4 conditions at the Friedrich-Loeffler-Institut.

### Virus infection and multicycle replication kinetics

Confluent SW13 cells were inoculated with NSDV or CCHFV at a multiplicity of infection (MOI) of 0.1. Supernatants and cell lysates were harvested at different times post infection (p.i.). Determination of viral titers was performed using plaque assay. Briefly, 10-fold serial dilutions of the virus stocks were on confluent SW13 cells. After 1 h, the inoculum was replaced by an Avicel-containing solid overlay (Carboxymethylcellulose sodium; DuPont). Fixation and crystal-violet staining were performed after 4 days of incubation.

### Plasmids and transfection of mammalian cells

For recombinant protein expression in insect cells, pAB-bee plasmids were designed to encode either the full-length NSDV nucleoprotein (NSDV N) or a GPC fragment (aa 138–445 including the putative NSDV GP38; _138_GPC_445_). Both sequences carry an N-terminal 6×histidine-tag and a FLAG-tag (DYKDDDDK) for protein purification (pAB-bee^TM^ NSDV N-his/FLAG and pAB-bee^TM^ NSDV GP38-his/FLAG). Sequences were codon-optimized for expression in insect cells and synthesized by Thermo Fisher Scientific. For recombinant protein expression in mammalian cells, a gene fragment from NSDV [IG619] GPC encoding aa 1–445 (_1_MLD-GP38_445_) was fused to a C-terminal human rhinovirus 3C 5HRV3C protease cleavage site and a Twin-strep-tag. This sequence was synthesized and codon-optimized for expression in human cells before subcloning into the mammalian expression vector pcDNA 3.1. (pcDNA NSDV MLD-GP38-Strep**;** Thermo Fisher Scientific). HEK-293T cells were transfected using polyethyleneimine (PEI) in a DNA-PEI ratio of 1:3. SW13 cells were transfected using Lipofectamine 3000 (Thermo Fisher Scientific) according to manufacturer’s instructions. To increase endogenous furin expression and to analyze the potential effect of furin on NSDV glycoprotein processing, HEK-293T and SW13 cells were transiently co-transfected with a plasmid encoding for a human furin protease (pIR_hFURIN_V5). All plasmid sequences are available upon request.

### Generation of recombinant baculoviruses for expression of NSDV GP38-his/FLAG and NSDV N-his/FLAG in Sf9 cells

pAB-bee NSDV N-his/FLAG or pAB-bee NSDV GP38-his/FLAG was co-transfected with baculovirus vector DNA (ProFoldER1 technology) into Sf9 cells using Profectin. Supernatants from transfected cells containing recombinant baculoviruses were harvested after an incubation period of four days at 27.5°C before viral titers were determined. For protein expression, Sf9 cells were infected at an MOI of 10 and incubated for 4 days at 27.5°C.

### His-tag purification from Sf9 cells

For the purification of NSDV GP38-his/FLAG and NSDV N-his/FLAG, baculovirus-infected Sf9 cells from 10× T175 flasks were harvested and centrifuged for 10 min at 1,000 × *g* at 4°C. After two washes with phosphate-buffered saline (PBS, 50 mM potassium phosphate, pH 7.2; 150 mM NaCl), cell pellets were resuspended in lysis buffer (170 mM NaCl, 10 mM imidazole, pH 8.0 with EDTA-free protease inhibitor cocktail “cOmplete,” Sigma-Aldrich) and then incubated on ice for 30 min. Lysates were clarified at 20,000 × *g* for 45 min at 4°C, and supernatant was retained for protein purification. NSDV GP38-his/FLAG and NSDV N-his/FLAG were purified by incubating the cleared supernatant overnight at 4°C with Ni-NTA Agarose (Superflow, Quiagen). Then, proteins bound to the agarose were pelleted and washed five times with 9 mL of washing buffer (50 mM PBS, 500 mM NaCl, 40 mM imidazole, 1% glycerol, pH 8.0) before elution of the protein using an elution buffer with increasing concentrations of imidazole per elution step (50 mM PBS, 500 mM NaCl, 100 up to 300 mM imidazole, 1% glycerol, pH 8.0).

### Monoclonal and polyclonal antibodies

Manipulations of animals at the Friedrich-Loeffler-Institut for the generation of monoclonal and polyclonal antibodies mentioned in this report were approved (LALLF M-V/TSD/7221.3-2.-042/17 and 7221.3-2-003/23) by the competent authority of the Federal State of Mecklenburg-Western Pomerania, Germany. Female BALB/c mice were immunized four times in an interval of 3 weeks intraperitoneally with 25 µg of recombinant NSDV GP38-his/FLAG or NSDV Gc (NAC-REC31906-500, The Native Antigen Company), mixed with an equal amount of GERBU Adjuvant MM (GERBU Biotechnik GmbH). Three days after the final boost, the immunized mice were euthanized and the spleens were removed under aseptic conditions. The generation of monoclonal antibodies was performed as described previously (54). Subcloning resulted in monoclonal antibodies (mAbs) 6F6 A3B raised against NSDV GP38-his/FLAG and 5H11 C1 raised against NSDV Gc. Both mAbs were used in immunoblot analyses.

A polyclonal rabbit-derived in-house antiserum raised against CCHFV N and cross-reacting with NSDV N (23) was used to stain NSDV N in immunoblot analysis (dilution of 1:1,000). Furthermore, we tested serum samples from *n* = 6 sheep infected with NSDV from a previous animal experimentation trial (12) in indirect ELISA in a dilution of 1:100.

### Immunoblot analysis

To determine the expression of different viral and cellular proteins in cell lysates, cells were lysed in 1% SDS in PBS. Sample loading buffer containing 40% glycerol, 0.1% bromophenol blue, 200 mM Tris (pH 6.8), and 4% β-mercaptoethanol was added to the sample. The proteins were separated in a 12% SDS-PAGE under reducing conditions with subsequent semidry Western blotting onto a nitrocellulose membrane. Blots were blocked with 5% skim milk powder in PBS. Proteins were stained using specific primary antibodies as outlined above with IRDye800 or 680 CW-conjugated goat anti-mouse or anti-rabbit IgG (LI-Cor Biosciences) or Horseradish peroxidase (HRP)-conjugated anti-mouse and anti-rabbit IgG (Invitrogen) as secondary antibodies. Blots were either imaged using the Li-Cor Odyssey CLx system or by chemiluminescence. Protein detection and quantification of signal intensity were performed using Image Studio Ver 5.2 (55). Molecular weights of protein bands were determined using Empiria Studio 3.2 software.

To determine the expression of viral and recombinantly expressed proteins in cell culture supernatants, samples from supernatant were either directly subjected to SDS-PAGE with subsequent immunoblot analysis as described above or concentrated using the Twin-Strep-tag and Strep-Tactin XT 4Flow before further analyses.

For deglycosylation of recombinant NSDV GP38-his/FLAG, heat-denatured recombinant protein was digested with PNGase F following manufacturer’s instructions (New England Biolabs), and samples were analyzed by SDS-PAGE followed by immunoblot as described above.

### Indirect immunofluorescence assay

iIFA was performed on NSDV-infected SW13 cells grown on glass coverslips. Briefly, after fixation with ice-cold methanol/acetone (1:1), cells were incubated with mAb 6F6 A3B for 1 h, followed by incubation with Alexa Fluor 488-conjugated secondary antibodies for 45 min. Cell nuclei were counterstained with 4′,6-diamidino-2-phenylindole (DAPI). Images were acquired with an Eclipse Ti-S inverted microscope system (magnification, 20×).

### Viability assay and compound usage in virus infection

Cell viability assay (MTT Assay, Roche) was performed in SW13 cells according to manufacturer’s instructions using different concentrations (10 µM and 30 µM) of furin inhibitor MI-1148 reconstituted in DMSO (37). To test the impact of furin on virus replication and glycoprotein processing, furin inhibitor MI-1148 was added to NSDV- and CCHFV-infected cells 1 h after virus inoculation. Cell lysates and supernatant were collected at 24 h p.i. and stored at −80°C until further processing. Samples were then either subjected to SDS-PAGE for further analysis in immunoblot or to plaque assay for determination of viral titers.

### In-house indirect ELISA for sheep based on NSDV GP38-his/FLAG or NSDV N-his/FLAG proteins

The recombinant proteins were coated (200 ng/well, diluted in 0.01 M PBS) on Greiner F-Medisorp 96 well plates and incubated overnight at 4°C. As a control for unspecific binding of the serum sample, a 6×histidine-tagged green fluorescent protein (GFP), also expressed in Sf9 cells, was coated in equal amounts (56). Plates were washed once with 250 µL washing buffer (PBS with 0.05% Tween20, Sigma-Aldrich; PBST) and blocked with blocking buffer (IDvet) for 1 h at 37°C. Each sheep serum sample was diluted 1:100 in IDvet Dilution Buffer No.14 and added in duplicate to the control- and antigen-containing wells (100 µL/well). After incubation for 1 h at 37°C, plates were washed three times with PBST before protein G peroxidase conjugate (MEMD Milipore) was added in a dilution of 1:5,000 in IDvet Dilution Buffer No. 3 and incubated for another 1 h at 37°C. After three washes with PBST, 3,3′,5,5′-tetramethylbenzidine (TMB) peroxidase substrate (Bio-Rad, Munich) was added. The reaction was stopped after 10  min at room temperature with equal amounts of 1 M sulfuric acid. Absorbance was measured at 450 against 620 nm in a Tecan Infinite 200Pro ELISA Reader (Tecan GmbH).

### In-house indirect ELISA based on NSDV Gn and Gc proteins

NSDV Gn (*NAC-REC31904-500*) and NSDV Gc (*NAC-REC31906-500*) from The Native Antigen Company were diluted in 0.01 M PBS and coated overnight on Maxisorp 96-well plates at a concentration of 100 ng/well. Plates were blocked with 5% skim milk in 0.01 M PBS at 37°C for 1 h and washed one time with PBST. Sera were diluted 1:100 in 2.5% skim milk in PBST and tested in duplicate. After 1 h incubation at 37°C, plates were washed three times with PBST and donkey anti-sheep IgG HRP conjugate (Jackson ImmunoResearch), diluted 1:20,000 in 2.5% skim milk in PBST, was added for 1 h at 37°C. Detection with TMB substrate and measurement was performed as described above.

### Mass spectrometry

In-gel digest was performed using standard procedures (57) with trypsin (Promega V5111) or Lys-C (Sigma P3428) as proteases. Peptide mixtures were desalted with C18 Zip tips (Millipore ZTC18S096) and analyzed on a nanoElute/timsTOF Pro LC-MS platform using the parameters suggested by the manufacturer (Bruker) for proteomic samples in data-dependent acquisition mode. A Bruker Pepsep Xtreme 25 cm column (Bruker #1893476) was used for separation with a binary gradient generated from 0.1% formic acid in water (solvent A) or in acetonitrile (solvent B), respectively. Elution was performed at 400 nL/min by raising B from 2% to 35% over 60 min. MS raw data were analyzed with the Fragpipe analytical platform (version 22, https://fragpipe.nesvilab.org) using a sequence database compiled from the Uniprot (58) human proteome (downloaded from https://www.uniprot.org/ on 02/20/2024) and sequences of the GP38 precursor and putative processing products originating from proteolytic cleavage at positions 175↓176 (a potential cleavage site identified by MS in preliminary experiments) and 134↓135 (a putative furin cleavage site suggested by the presence of an N-terminal RVGR [aa 131–134] motif). A maximum of two missed cleavage sites were tolerated. Protein N-terminal acetylation and methionine oxidation were allowed as optional modifications, and cysteine residues were set to be carbamidomethylated. Database queries with Fragpipe were conducted with both full enzyme specificities and with semi-specific enzyme specificities.

### Synthesis of fluorescence resonance energy transfer substrates

The peptides were synthesized by a standard Fmoc solid phase peptide synthesis protocol using 2 mL reaction vessels in a multiple peptide synthesizer Syro 2000 (MultiSynTech GmbH, Witten, Germany). For the synthesis, approximately 120 mg of an Fmoc-Rink-MBHA-amide resin (IRIS Biotech, loading 0.57 mmol/g) was used, which was initially loaded with Fmoc-Tyr(3 NO_2_)-OH. All coupling reactions were performed twice (double couplings) for 30 min with an approximately fourfold excess of Fmoc amino acid, HCTU, respectively, and 8 equiv. NMM, as described previously (59). After each coupling cycle, the remaining free amino groups were capped with acetic anhydride. After the final coupling cycle with 2-(Boc-NH)-benzoic acid, the resin was additionally treated with 20% piperidine in DMF to remove any potential acylation on the 3-nitrotyrosine side chain (60). The peptides were cleaved from the resin and deprotected by a solution of TFA/triisopropylsilane/water (95/2.5/2.5; vol/vol/vol) for 5 h and precipitated in cold diethyl ether. All crude peptides were purified by preparative reverse-phase HPLC to more than 95% purity based on the detection at 220 nm and finally obtained as lyophilized TFA salts.

Further information regarding analytical high-performance liquid chromatography (HPLC) and MS experiments is provided in Table S1.

### Enzyme kinetic measurements with recombinant furin

The measurements with the synthesized FRET substrates were performed in black 96-well plates (Nunc) at room temperature with a microplate reader (Spark, Tecan Group Ltd., Männedorf, Switzerland) at *λ*ex 320 nm and *λ*em 420 nm (61). Each well contained 20 µL of the substrate solution (dissolved in water) and 150 µL buffer (100 mM Hepes, 0.2% Triton X-100, 2 mM CaCl_2_, 0.02% sodium azide, and 1 mg/mL BSA, pH 7.0). The measurements were started by the addition of 20 µL furin solution (0.758 nM in assay, kindly provided by Iris Lindberg, University of Maryland, Baltimore, MD [62]). The measurements were performed over a period of 10 min, and the steady-state rates were calculated from the slopes of the progress curves.

### Data and sequence analysis

Figures were created with GraphPad Prism (version 9.3.1). Sequence alignment was generated with Clustal Omega using Geneious prime software (version 2021.0.1) with the following accession numbers for the GPC sequences: Ganjam virus IG619 (GenBank accession number EU697950), hereafter referred to as NSDV_India, CCHFV IbAr10200 (GenBank accession number U39455.2), NSDV strain Jilin (referred to as NSDV_China; accession number NC_034391), and NSDV strain 708 (referred to as NSDV_Kenia; accession number EU697952). The visualization was performed with ESPript 3.x (63).

## SUPPLEMENTAL MATERIAL

Supplemental figures jvi.00537-25-s0001.pdf Figures S1 to S5.

Table S1 jvi.00537-25-s0002.pdf Sequences and analytical data of the GPC-derived substrates.

ASM does not own the copyrights to Supplemental Material that may be linked to, or accessed through, an article. The authors have granted ASM a non-exclusive, world-wide license to publish the Supplemental Material files. Please contact the corresponding author directly for reuse.
