# Supplementary material for: Immunogenicity of NSDV GP38 and the role of furin in GP38 proteolytic processing
Source: J Virol. 2025 Jun 10;99(7):e00537-25. doi: 10.1128/jvi.00537-25 (PMC12282141; doi:10.1128/jvi.00537-25)
Supplement: Supplemental figures — Figures S1 to S5. [file jvi.00537-25-s0001.pdf]

Supplementary Material

Supplementary Figure 1

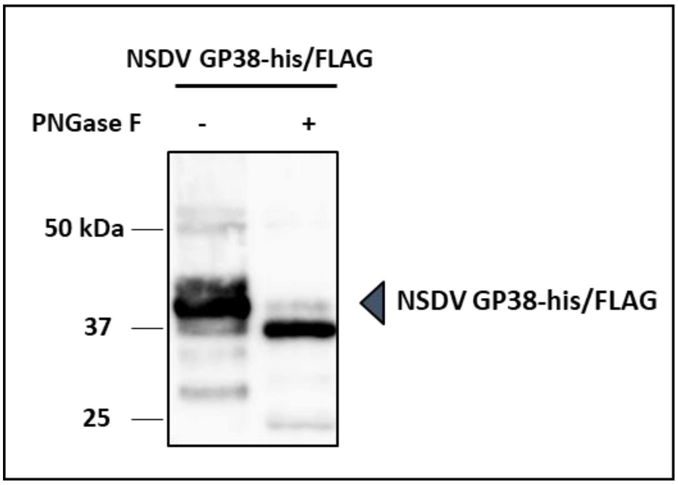

Suppl. Fig. 1: PNGase F digestion of recombinant NSDV GP38-his/FLAG analyzed by immunoblot. SDS-PAGE of recombinant NSDV GP38-his/FLAG his-tag-purified from Sf9 cells was followed by immunoblot analysis using anti-FLAG primary and HRP-conjugated secondary antibodies.

Supplementary Figure 2

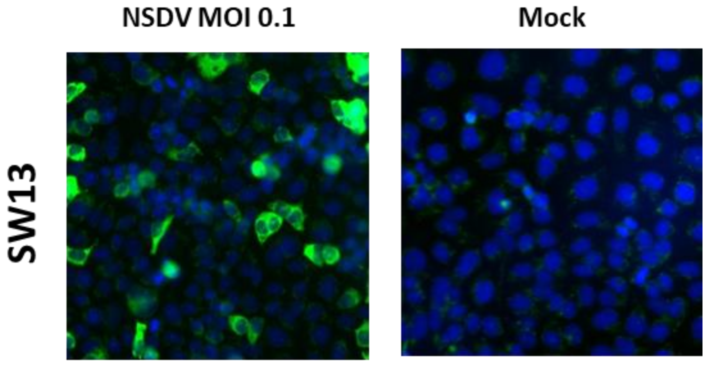

Suppl. Fig. 2: Characterization of monoclonal antibody (mAb) 6F6 A3B raised against recombinant NSDV GP38-his/FLAG. Immunofluorescence analysis of NSDV-infected and mock-infected SW13 using mAb 6F6 A3B. Cells were fixed using methanol/acetone at 24 h post infection (p.i.).

Supplementary Figure 3

3A

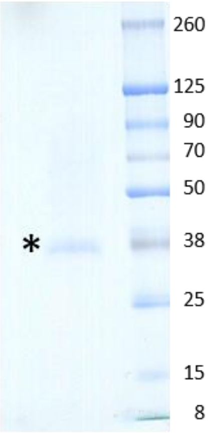

3B

M A L V A K G L I L M L L L Y M F K A S E V N L E L P S W N S T E G T E Y A D E L P T E P P S E
E E P K L A A V S T V T T P A P P K E S T E D T I E S P D G G E T M A P T T P A P V T D S A S K
L E E E P G T T Q Q A Q S T Q M Q N S Q A P I G M P E A W S T S P S R V G R K P L S A S P D V S
E P D Q N T E A Q T H P P V F L D G R P V E E E E H S V G T R D V A S D L R E Y N Q M K Q L L S
Q R I L A K M G M A V D F T D D E L D T W C Y R R Y S N C S S N D I E S R I R D F F L I T D R S
E C F D E V L V K R L C E T T S P I I D R A W K I A G L K E E V V L R E M G R R I F R F F T P A
L K V T C M S G S L N P S N Q F V R F Y N P T L E R T S G P T I Q N Y D G M H C L N I E N G L I
K P S K V V V I N V L M T T V D I R L E S C R A F I N A Q Q C T Y T Q H A D G L V R V P T F V G
P H G E K R I I G A Y T M S F N L T D E K N K A C T I K T T C V V K G K E V K K G Q S Q L R G F
P T T I R L F K S V T G K L E V L F Q G P A A A S W S H P Q F E K G G G S E G G S G G G S W S H
P Q F E K

M A L V A K G L I L M L L L Y M F K A S E V N L E L P S W N S T E G T E Y A D E L P T E P P S E
E E P K L A A V S T V T T P A P P K E S T E D T I E S P D G G E T M A P T T P A P V T D S A S K
L E E E P G T T Q Q A Q S T Q M Q N S Q A P I G M P E A W S T S P S R V G R K P L S A S P D V S
E P D Q N T E A Q T H P P V F L D G R P V E E E E H S V G T R D V A S D L R E Y N Q M K Q L L S
Q R I L A K M G M A V D F T D D E L D T W C Y R R Y S N C S S N D I E S R I R D F F L I T D R S
E C F D E V L V K R L C E T T S P I I D R A W K I A G L K E E V V L R E M G R R I F R F F T P A
L K V T C M S G S L N P S N Q F V R F Y N P T L E R T S G P T I Q N Y D G M H C L N I E N G L I
K P S K V V V I N V L M T T V D I R L E S C R A F I N A Q Q C T Y T Q H A D G L V R V P T F V G
P H G E K R I I G A Y T M S F N L T D E K N K A C T I K T T C V V K G K E V K K G Q S Q L R G F
P T T I R L F K S V T G K L E V L F Q G P A A A S W S H P Q F E K G G G S E G G S G G G S W S H
P Q F E K

3C

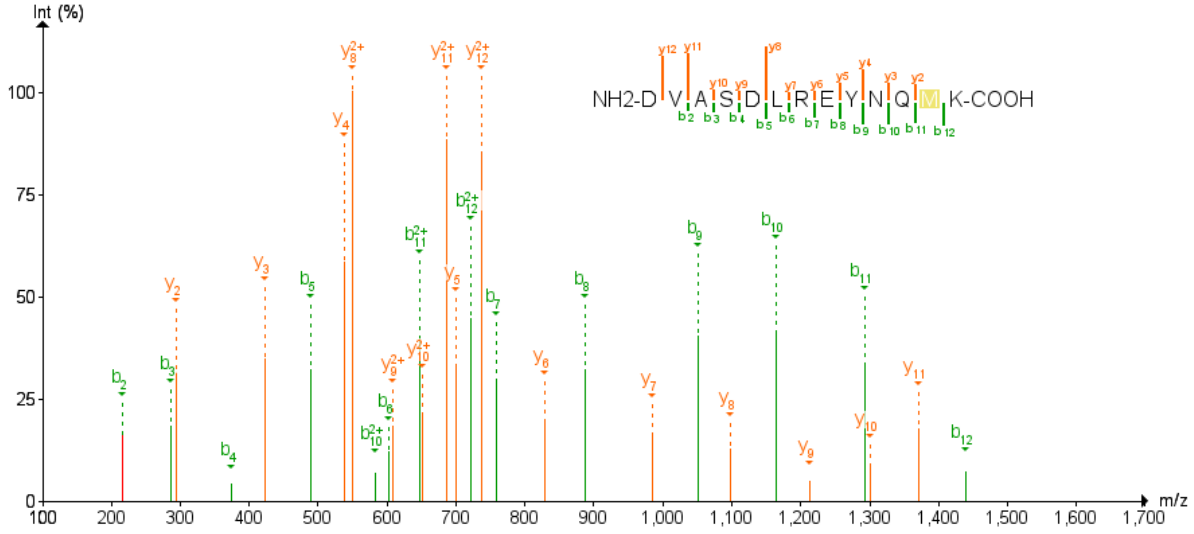

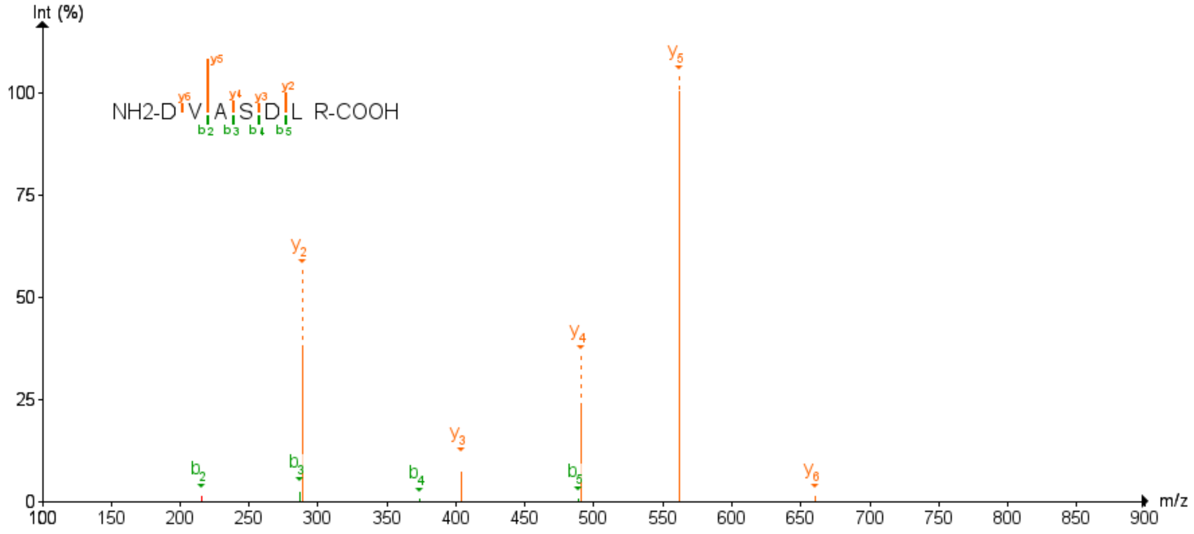

Suppl. Fig. 3: A. Samples of the processed GP38 protein were purified and separated by SDS PAGE. A star indicates the band that was selected for in-gel digest and MS analysis. Marker masses are indicated in kDa. B. The upper and lower panels show the coverages (green) of the GP38 precursor sequences by peptides identified after digest with Lys-C and trypsin, respectively. C. The upper and lower panels show the fragment spectra (b- and y-ion series only) of the peptides starting at position 176 of the GP38 precursor protein generated by in-gel digest of the 38 kDa processing product using Lys-C (DVASDLREYNQMK) or trypsin (DVASDLR), respectively. Nearly complete y- and b-ion series were obtained confirming the

sequence. Note that Lys-C does not cut C-terminal of R, indicating that position 176 was the N-terminus of the processed protein prior to the digest with Lys-C. In contrast, trypsin does cut after R, producing the shorter peptide DVASDLR with the fragmentation pattern depicted in the lower panel.

Supplementary Figure 4

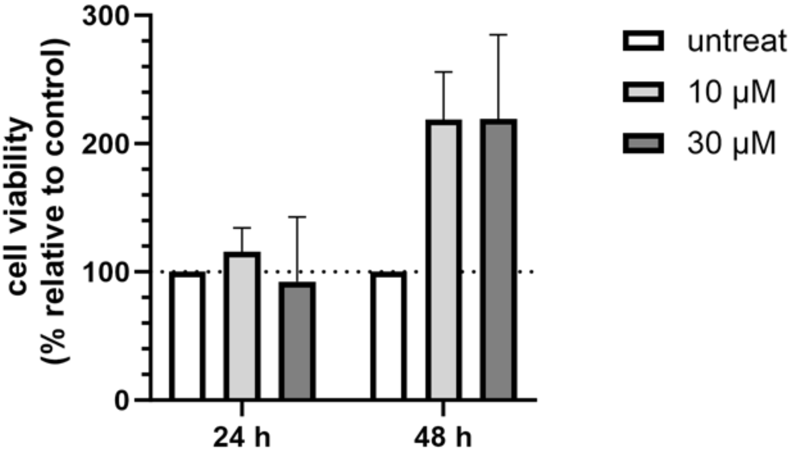

Suppl. Fig. 4: Cell viability of SW13 cells after treatment with inhibitor MI-1148 for 24 and 48 h. Cell viability is expressed as percentage of the control group. Two independent experiments were performed in quadruplicate each.

Supplementary Figure 5

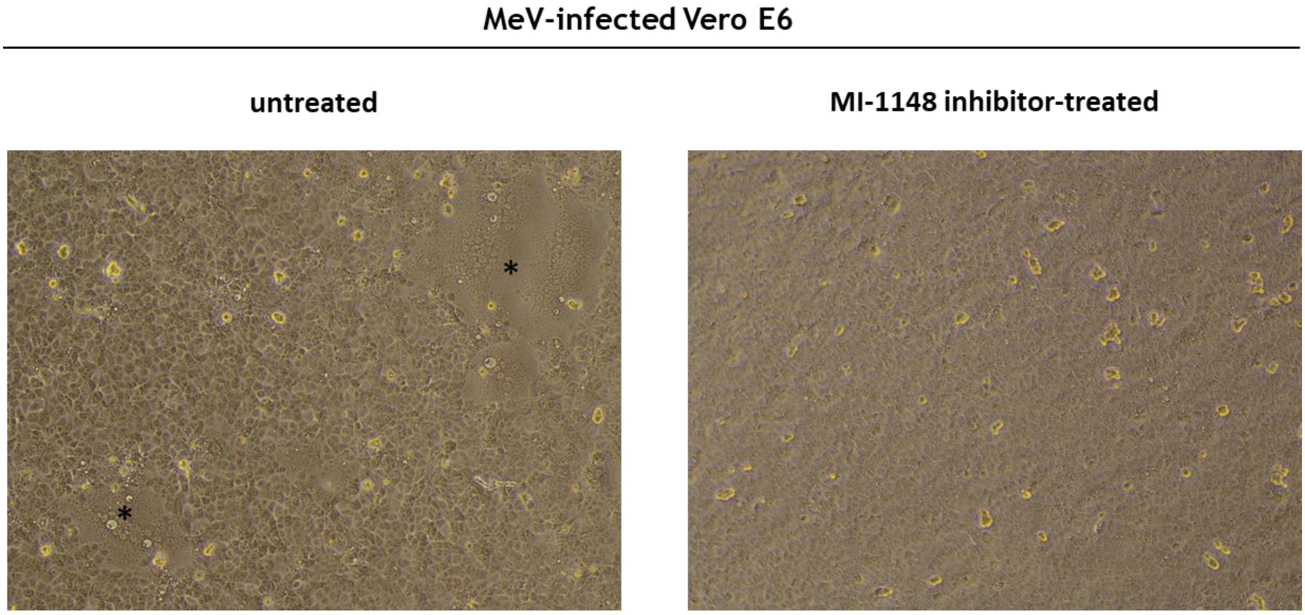

Suppl. Fig. 5: Vero E6 cells were inoculated with Measles virus (MeV; MOI 0.2) and treated with inhibitor MI-1148 or left untreated at 1 h post inoculation. Pictures were captured at 36 h post infection. Asterisks indicate syncytia formation.
