# Supplementary material for: Immunogenicity of NSDV GP38 and the role of furin in GP38 proteolytic processing
Source: J Virol. 2025 Jun 10;99(7):e00537-25. doi: 10.1128/jvi.00537-25 (PMC12282141; doi:10.1128/jvi.00537-25)
Supplement: Table S1 — Sequences and analytical data of the GPC-derived substrates. [file jvi.00537-25-s0002.pdf]

Supplementary Table 1. Sequences and analytical data of the GPC-derived substrates.

Peptide	Sequence	HPLC (% solvent B)	MS calc.	MS found
GPC-1	Abz-EHSVGTRDVAS-Tyr(NO_2)-NH_2	27.5	1482.65	742.96 $(M+2H)^{2+}/2$
GPC-2	Abz-EHSVGTADVAS-Tyr(NO_2)-NH_2	29.9	1397.58	700.40 $(M+2H)^{2+}/2$
GPC-3	Abz-SPSRVGRKPLS-Tyr(NO_2)-NH_2	27.3	1509.77	1511.00 $(M+H)^{+}$

Abz = 2-aminobenzoic acid

All analytical HPLC experiments were performed on an Agilent 1260 Inf II system (Agilent Santa Clara, CA, USA; column: NUCLEODUR C18 ec, 5 µm, 100 Å, 4.6 mm x 250 mm, Macherey-Nagel, Düren, Germany) with 0.1 % (*v/v*) TFA in water (solvent A) and 0.1 % (*v/v*) TFA in acetonitrile (solvent B) as eluents. A linear gradient with an increase of 1 % solvent B/min at a flowrate of 1 mL/min was applied. Purification of the peptides was performed by preparative HPLC using a Knauer system (Knauer Azura P 2.1L with HyperShear static mixer; detector: Knauer UVD 2.1L Berlin, Germany; collector Foxy R1, column: NUCLEODUR C18 ec, 5 µm, 100 Å, 32 mm x 250 mm, Macherey-Nagel, Allentown, PA, USA) with the same solvents as used for the analytical experiments. In this case, a linear gradient with an increase of 0.5 % solvent B/min was applied. Detection for both analytical and preparative experiments was performed at 220 nm. Mass spectrometry was performed on a QTrap 2000 ESI spectrometer (Applied Biosystems, Norwalk, CT, USA).
